# An international classification of functioning, disability and health-children and youth (ICF-CY) based assessment tool for evaluating impairments, function and participation of children and adolescents with cerebral palsy in special education schools

**DOI:** 10.12669/pjms.42.6.16682

**Published:** 2026-06

**Authors:** Roohi Abbas, Muhammad Naveed Babur, Saleh Shah, Saba Awan

**Affiliations:** 1 Dr. Roohi Abbas, PT, MSNMPT, Department of Physical Therapy, Faculty of Allied Health Sciences, Superior University Lahore, Lahore, Pakistan; 2 Prof. Dr. Muhammad Naveed Babur, PT, PhD, Department of Physical Therapy, Faculty of Allied Health Sciences, Superior University Lahore, Lahore, Pakistan; 3 Dr. Saleh Shah, PT, PhD, Rehabilitation Sciences, Department of Physical Therapy, Faculty of Allied Health Sciences, Superior University Lahore, Lahore, Pakistan; 4 Dr. Saba Awan, PT, DPT, Physiotherapist, Govt. In Service Training College for the Teachers of Disabled Children, Lahore, Pakistan

**Keywords:** Classification of Functioning, Cerebral palsy, Classification, Functioning, Disability and Health, International outcome assessment, ICF-CY, Special education

## Abstract

**Objectives:**

To develop an International Classification of Functioning, Disability and Health-Children and Youth (ICF-CY) based questionnaire for evaluating impairments, function, and participation of children and adolescents with cerebral palsy (CP) in special education schools, and to evaluate the content validity and inter-rater reliability of the questionnaire

**Methodology:**

An ICF-CY(6-14 years) based, 28 items assessment tool for CP was developed in the first phase of the study by three experts. Content validity and inter-rater reliability of the questionnaire were evaluated in the second & third phases. SPSS version 14 was used for data analysis. Descriptive statistics (Mean ± SD) were calculated for total scores by age, gender, type of CP, grade/class, hand dominance, and history of epilepsy. Content validity was determined by calculating Item-CVI and Scale-CVI, while Inter-rater reliability was determined using two-way mixed-effects intraclass correlation coefficients (ICC, consistency, single measure). It was carried out in two special education schools affiliated with the Directorate of Special Education, Lahore, from May 2025 to January 2026.

**Results:**

Average I-CVI (0.9069 to 0.9438) were greater than the recommended threshold of 0.78, indicating excellent item-level validity. ICC ranged from 0.751 to 0.989, with a 95% confidence interval indicating moderate to high Reliability.

**Conclusion:**

This preliminary study shows that the ICF-CY-based assessment tool for CP has good content validity and reliability and can be used to carry out routine functional assessment in special education schools.

## INTRODUCTION

International Classification of Functioning, Disability and Health (ICF model)-based assessment tools are gaining significant importance in identifying and classifying the functional status of individuals with certain health conditions.^1^ The World Health Organization, in its effort to regulate the description of functioning and dysfunction, proposed and created a reference family of classifications based on ICF) model.^2^ International Classification of Functioning, Disability and Health-Children and Youth version (ICF-CY) is exclusively developed to cater special needs of children and youth with cerebral palsy as the original ICF model is very comprehensive and not feasible to implement in routine examination. Plenty of international literature recognizes the importance of using ICF-based assessment measures to document goal-setting, intervention planning and measuring functioning profile of patients with neurological conditions such as Cerebral Palsy.^3^

Cerebral palsy (CP) is a generic expression for disorders related to posture and mobility, not precisely defined and lacks internationally standardized inclusion criteria.^4^ Permanent damage to premature brain leads to characteristic motor disorders along with turbulences of cognition, perception, sensation, communication, emotional & behavioral issues.^5^ This heterogeneity in clinical presentation of cerebral palsy needs an extensive, holistic, and personalized approach to understanding and assessing the unique requirements of every CP patient to improve their function and quality of life.^6^

From a physiotherapy and rehabilitation perspective, dealing with cerebral palsy, just like any other neurological disorder is very challenging. In routine examination, the evaluation of motor disorders in improving upper or lower extremity function is emphasized, but the literature supports the evaluation of the nonmotor component of dysfunction as an integral part of routine examination.^7^ Several performance-based clinical evaluation questionnaires have been developed based on the ICF model to provide a more structured analysis of almost every aspect of functioning and dysfunction.^8^

Since the emphasis in CP patients is to improve performance and make them as independent as possible, the assessment generally revolves around detecting what the child can’t do. A recent systematic review endorses ICF-CY as a framework for understanding & documenting health status, functioning, activity/participation, and influence of CP on patients, particularly for Physical Therapists.^9^ Another study by Polo et al in 2009 used ICF-CY based questionnaire to make “Functional Diagnosis” of a child with cerebral palsy of school-going age, entailing the problems in body structure/function, restrictions in performance as well as the potential of the child to do certain tasks and activities which is the essence of ICF model .^10^ The routine assessments in our clinical and academic settings lack standardization and documentation with very limited utilization of ICF model and its related versions. The only and recent study from Pakistan by Hina et al has endorsed use of ICF-CY brief core set as standardized assessment tool to identify functional level of cerebral palsy children in hospital setting.^11^ Moreover, only one local evidence on the significance of ICF utility in special education was presented by Shaheen Pasha in 2009, who not only highlighted the lack of standardized assessment in special education but strongly recommended adaptation of ICF framework to improve quality of education and quality of life of children with special needs.^12^ Hence, a universal language for assessment that can be documented and utilized academically and clinically is of utmost importance.^13^

This Study aims to develop an ICF-CY-based questionnaire for evaluating impairments, function, and participation of children and adolescents with cerebral palsy in special education schools, and to evaluate the content validity and inter-rater reliability of the questionnaire.

## METHODOLOGY

It was a multi-phase assessment tool development and validation study approved by the local institutional review board (IRB/FAHS/REHAB/06/11/PhD/RS-4633; dated April 17, 2025). Study comprised an Initial qualitative phase of item generation based on literature review and meetings with special education teachers and a quantitative phase of psychometric evaluation, using a cross-sectional study design with purposive sampling. It was carried out in two special education schools affiliated with the Directorate of Special Education, Lahore, from May 2025 to January 2026. Children and Adolescents (6-14years) with cerebral palsy enrolled in special education schools under “physical disability” were included, and those having severe cognitive dysfunction or any other neurological condition other than CP were excluded from the study.

### Participants:

The first phase of item generation of the questionnaire was done by three experts. The second phase of content validity involved eight subject experts, and the final phase assessing inter-rater reliability involved three raters evaluating 19 children and adolescents with cerebral palsy (15 males, 4 females; mean age 9.2±2.3 years).

### Development of the ICF-CY (6-14 years) based questionnaire for children and adolescents with cerebral palsy:

The brief ICF-CY core set for CP (6-14 years), having 35 ICF categories were analyzed for relevant item generation and initial draft was formulated. 12 Items for Body function (b), 13 for Activity and participation (d) and one item for environment (e) was added.^14^ ICF qualifiers were used to rate each item of the questionnaire from 0 to 4 where 0 indicates no problem and 4 indicates complete problem as 0=No problem(0-4%) 1=Mild problem(5-24%) 2=Moderate problem(25-49%) 3=Severe Problem(50-95%) 4=Complete problem(96-100%).^15^,^16^

### Expert Validity:

The questionnaire was assessed by three experts (two special education teachers and one physiotherapist). The experts deleted one of the items from body function (b) which was not relevant to special education context, and added three more sub-items in activity and participation domain(d) making it a 28-item questionnaire. Now the domain body function (b) contain 11 items covering intellectual function (b117), attention function(b140), motivation(b131), Mental functions of language(b167), Seeing functions(210), Sensation of pain(b280), Mobility of joint functions (b710), Muscle tone functions (b735) and Control of voluntary movement functions (b760). The Activity & participation(d) domain include 16 items covering Solving problems(d175 ), Carrying out daily routine(d230), Conversation(d350), Maintaining a body position(d415), Fine hand use(d440), Walking(d450), Moving around in different locations(d460), Toileting(d530), Eating(d550), Basic interpersonal interactions(d710), Family relationships(d760), School education(d820) and, Recreation and leisure(d920). The contextual factor of Environment (e) include one item covering Products and technology for personal use in daily living(e115).

### Data collection:

Two Physiotherapists collected data using questionnaire based on ICF-CY brief core set with 28 items. The raters were first given instructions and guidelines regarding the ICF-CY-based questionnaire. Written informed consent was taken from the participants and parents/caregivers of CP child.

### Statistical analysis:

Data Analysis was done using SPSS version 14. Descriptive statistics (Mean ± SD) were calculated for total scores by age, gender, type of CP, grade/class, hand dominance and history of epilepsy. The content validity of the 28-item questionnaire was determined by rating each question by eight experts (special education teachers, > 5 years CP experience, mean = 13.5 years) on criteria of relevance, clarity, simplicity and ambiguity using 4-point Likert scale, rating was then dichotomized to calculate Item-CVI and Scale-CVI. Inter-rater reliability was analyzed using two-way mixed-effects Intraclass correlation coefficients (ICC; consistency, single measure) with 95% confidence intervals.

## RESULTS

The Demographics and clinical characteristics of Children and adolescents with cerebral palsy who participated in our study are shown in Table-I. The nineteen participants (15 males and four females) had a mean age of 9.2±2.3 years. Most of them had a spastic type of CP (78.9%) with right-sided hand dominance (78.9%) and no history of epilepsy (84.2%) (Table-I).

**Table-I:** Demographics and clinical characteristics of children and adolescents with cerebral palsy (n=19).

Characteristics	
Chronological age n (%)	Mean(SD)
6 to 8 years	6 (31.6)
9 to 11 years	7 (36.8)
12 to14 years	6 (31.6)
***Gender n (%)***	
Male	15 (78.9)
Female	4 (21.1)
***Type of CP n (%)***	
Spastic	15 (78.9)
Dyskinetic	1 (5.3)
Mixed	3 (15.8)
***Grade/Class n (%)***	
Below grade 1	4 (21.1)
1 to 5	12 (63.2)
5 to 10	3 (15.8)
***Dominance n (%)***	
Right handed	15 (78.9)
Left handed	3 (15.8)
No developed	1 (5.3)
***Epilepsy History n (%)***	
No	16 (84.2)
Yes	3 (15.8)

The Content validity Index (CVI) of the questionnaire was calculated using Scale level-CVI and Item level-CVI based on expert ratings across 28 items for relevance, clarity, simplicity & ambiguity. Average I-CVI (0.9069 to 0.9438) were greater than recommended threshold of 0.78, indicating excellent item-level validity. The Scale-CVI (S-CVI/Relevance: 0.9438; S-CVI/Clarity: 0.9069; S-CVI/Simplicity: 0.9365; S-CVI/Ambiguity: 0.9238) calculated as average of Item-CVI per subscale were also high, confirming robust scale level content validity. The Item-CVI analysis provided more specific details, most items achieved I-CVI = 1.00 across subscales, except for item three (clarity: 0.56); item 16 (ambiguity:0.56) indicating a need for a prospective change in items and based on this feedback, the wording of Items 3 and 16 was refined to enhance clarity and reduce ambiguity before subsequent data collection, in line with best practices for instrument development.^17^

Inter rater reliability analysis demonstrated excellent reliability across all domains of the ICF-CY-based questionnaire. Values for ICC ranged from 0.751 to 0.989 with 95% confidence interval. Tabl- 2 shows the mean scores and standard deviations were consistent among rater A, B and C for each item. The lowest reliability (ICC = 0.751, 95% CI), was found to be in the domain “Products and technology for personal use in daily living (E115)”, and the highest reliability (ICC = 0.989, 95% CI) was found to be in the domain “solving problems (dI175)”. Least impairment in body functions (b) 0.32±0.478 was found to be in intellectual function (b117) and mental function of language (b167) and the highest impairment in body function (b) 2.26±1.195 was found to be in muscle tone function (b735). In Activity and participation domain (d), the least impairment 0.63±0.895 was found in “family relationships (d760) and the highest impairment 2.16±1.214 was found in “Carrying out daily routine (d230)” and 2.16±1.119 in “Toileting (d530)”.

**Table-II:** Inter rater reliability of ICF-CY-based questionnaire (6 to 14 years) for children & Adolescents with cerebral palsy (n=19)

Domain/Categories	ICF code	Rater A	Rater B	Rater C	ICC(95% CI**)
		Mean(SD)	Mean(SD)	Mean(SD)	
Intellectual functions	b117	0.37(0.585)	0.32(0.478)	0.425(0.587)	0.838(0.690-0.928)
Motivation	b131	1.235(0.965)	1.05(0.970))	1.00(1.00)	0.982(0.961-0.992
Attention functions	b140	1.42(0.769)	1.50(0.707)	1.37(0.684)	0.960(0.914-0.983
Mental functions of language	b167	0.37(0.597)	0.32(0.478)	0.32(0.478)	0.944(0.885-0.976)
Seeing functions	b210	1.21(1.437)	1.33(1.534)	1.16(1.463)	0.975(0.946-0.990)
Sensation of pain	b280	0.63(0.895)	0.58(0.902)	0.58(0.902)	0.978(.954-0.991)
Mobility of joint functions	b710	1.95(1.471)	2.05(1.393)	2.11(1.410)	0.964(0.924-0.985)
Muscle tone functions	b735	2.21(1.182)	2.11(1.197)	2.26(1.195)	0.988(0.973-0.995)
Control of voluntary movement functions	b760	2.05(1.177)	2.21(1.032)	2.00(1.00)	0.866(0.739-0.941)
Solving problems	d175	1.58(1.261)	1.52(1.09)	1.63(1.212)	0.989(0.976-0.995)
Carrying out daily routine	d230	2.16(1.214)	2.16(1.259)	2.16(1.302)	0.965(0.927-0.985)
Conversation	d350	1.11(0.737)	1.32(0.885)	1.21(0.855)	0.855(0.720-0.936)
Maintaining a body position	d415	1.52(1.112)	1.76(1.361)	1.605(1.172)	0.932(0.862-0.971)
Fine hand use	d440	1.68(1.157)	1.68(1.156)	1.63(1.165)	0.987(0.972-0.995)
Walking	d450	1.95(1.129)	1.95(1.129)	1.89(1.197)	0.987(0.972-0.994)
Moving around in different locations	d460	2.05(1.129)	2.00(1.155)	2.00(1.202)	0.973(0.944-0.989)
Toileting	d530	2.16(1.119)	2.00(1.106)	2.11(1.150)	0.932(0.862-0.971)
Eating	d550	1.265(0.848)	1.18(0.845)	1.155(0.856)	0.952(0.900-0.979)
Basic interpersonal interactions	d710	0.74(0.806)	0.74(.806)	0.79(0.855)	0.974(0.945-0.989)
Family relationships	d760	0.63(0.895)	0.63(0.895)	0.68(0.885)	0.978(0.953-0.991)
School education	d820	0.89(0.809)	0.95(0.848)	0.89(.809)	0.974(0.945-0.989)
Recreation and leisure	d920	1.58(1.121)	1.53(1.172)	1.37(1.065)	0.867(0.740-0.941)
Products and technology for personal use in daily living	e115	1.42(0.92)	1.26(0.806)	1.37(0.955)	0.751(0.551-0.885)

## DISCUSSION

The ICF-CY provides a strong foundation for instrument and tool development in disability rehabilitation, but concerns about psychometric suitability are yet to be addressed.^18^,^19^ Rojas et al refers a study by Karlsson E et al. in 2021 highlighted the urgent need to validate ICF core sets in non-European countries from both the patient and professional perspectives.^18^-^20^ Our study is in line with rigorous research for implementing ICF framework encompassing preliminary development & validation of assessment tool based on ICF-CY.^21^ The scores for content validity index (CVI) of questionnaire from our study ranged between 0.9069 to 0.9438 for majority of items showing coherent and relevant items of questionnaire. However, two items fall at 0.56 which were further amended to use in prospective study which is quite similar to study by Gomes et al, which included content validation of a tool based on ICF-CY for persons with physical disabilities.^22^ The utility of ICF and its core sets based assessment in the educational context is imperative to establish an interprofessional and interdisciplinary collaboration for individualized interventions personalized to each child’s inimitable strengths and challenges. The ICF-CY-based questionnaire for use in special education used in our study showed excellent inter-rater reliability across all ICF-CY domains as ICC for 18 domains out of total 23 domains of ICF show exceptional agreement among raters, the lowest reliability were in the domain of “environment” which aligns with findings from other researchers.^23^ Environment (e) is the least addressed category of ICF in majority of the outcome measures negatively impacting real-life experiences of children and adolescents with cerebral palsy, emphasizing the need to add more items related to this category of ICF-CY in the assessment tools and questionnaires.^24^,^25^

Although ICF-CY has long been doubted for its relatively low validity and reliability as compared to other outcome measures, it can be used as a quick screening or basic assessment tool to profile the functioning level of children and youth with various disabilities.^26^

Therefore, utilization of ICF framework particularly ICF-CY can foster early individualized intervention plans (IIP) and individualized education plans (IEP) by empowering professionals (educators, physiotherapists, occupational therapists) to evaluate strengths, limitations and challenges of a child or adolescent with cerebral palsy in educational context.

ICF framework is implemented globally as a promising approach towards achieving rehabilitation goals and extensive research is in progress but there is a limited evidence on the adoption of this holistic model from developing countries like Pakistan. The only research work on the adoption of ICF-CY from Pakistan is from a hospital setting.^11^ The awareness and knowledge possessed by health care professionals in our settings regarding ICF framework and related domains is yet to be established, our study in this regard will be a benchmark in the implementation of a universal language and classification of ICF in the educational context to assess and document the functional profile of children and adolescents with cerebral palsy.^18^

The only limitation incurred is the heterogeneity in CP presentation across motor, sensory, behavioral and cognitive. The wide variability in motor, sensory, cognitive, and behavioral impairments among children with CP can affect the instrument’s sensitivity and specificity across different subgroups.

### Limitations:

Although the questionnaire has been validated for its content and inter-rater reliability, it doesn’t appraise its ability to measure the underlying construct, which is intended to measure, i.e., construct validity. This necessitates further validation.

## CONCLUSION

This preliminary study shows that the ICF-CY-based assessment tool for CP has good content validity and reliability and can be used to carry out routine functional assessment in special education schools. However, further psychometric evaluation of the questionnaire is recommended.

### Authors Contribution:

**RA:** conceived, designed and did statistical analysis

**NB:** Critical review, and final approval of manuscript

**SS:** Supervision, proofreading, and editing of the manuscript

**SA:** Literature search, data collection and manuscript writing

All Authors are responsible for integrity of the research.
